# A new decompression technique for upper lumbar fracture with neurologic deficit-comparison with traditional open posterior surgery

**DOI:** 10.1186/s12891-019-2897-1

**Published:** 2019-12-01

**Authors:** Bangke Zhang, Fengjin Zhou, Liang Wang, Haibin Wang, Jiayao Jiang, Qunfeng Guo, Xuhua Lu

**Affiliations:** 1 Department of Orthopaedics, Shanghai Changzheng Hospital, Second Military Medical University, Shanghai, China; 2 Department of Spinal Surgery, Xi’an Zhongde Orthopedics Hospital, Xi’an, China

**Keywords:** Upper lumbar fracture, Neurological deficit, Decompression, Paraspinal muscle, New surgical approach, Traditional posterior approach

## Abstract

**Background:**

Surgery is usually recommended for thoracolumbar fracture with neurologic deficit. However, traditional open posterior approach requires massive paraspinal muscles stripping, and the canal decompression may be limited and incomplete. We aimed to investigate a new approach via the Wiltse approach and the Kambin’s Triangle.

**Methods:**

Twenty-one consecutive patients with traumatic upper lumbar fracture who received this new approach surgery between January 2015 and January 2016 constituted the new approach group. Twenty-nine patients received the traditional open posterior surgery between January 2014 and January 2015 were classified as the traditional posterior surgery group. Surgical informations including operative time, blood loss, drainage volume, hospitalization days were collected and compared among the two groups. The American Spinal Injury Association (ASIA) impairment scale and Visual Analog Score (VAS) were evaluated preoperatively, postoperatively and at 12 months follow-up.

**Results:**

Patients in the new approach group had fewer operation time (128.3 ± 25.1 vs 151 ± 32.2 min, *P* = 0.01), less blood loss (243.8 ± 135.5 vs 437.8 ± 224.9 ml, *P* = 0.001) and drainage volume (70.7 ± 57.2 vs 271.7 ± 95.5 ml, *P* < 0.001), as well as shorter hospitalization stay than the traditional posterior surgery group (6.6 ± 1.8 vs 8.5 ± 2.4 d, *P* = 0.004). Similar neurologic recovery according to ASIA grade was achieved in both groups (Recovery index: 0.90 ± 0.53 vs 0.86 ± 0.51, *P* = 0.778). While the pain level was significantly lower in the new approach group postoperatively (2.6 ± 0.7 vs 3.5 ± 0.9, *P* < 0.001) and at 12 months follow-up (1.4 ± 0.9 vs 2.4 ± 0.8, *P* < 0.001).

**Conclusion:**

The present new approach was successfully applied in the treatment of upper lumbar fracture with neurologic deficit. It can reduce iatrogenic trauma and achieve similar or better outcomes compared to the traditional posterior surgery.

## Background

Almost 70% of traumatic spine fractures occur in the thoracolumbar region due to the great biomechanical stress in this area [1, 2], and 10 to 20% of these injuries are burst fractures [3]. Burst fractures can be 2 or 3 column injuries that may result in failure to support the anterior and middle column, and retropulsion of bone fragments into the spinal canal with consequent neurologic injury [4]. It is reported that more than a quarter of thoracolumbar burst fractures would lead to complete or incomplete neurologic deficit [1, 2] and operative treatment is usually recommended for these patients [5, 6]. Various surgical procedures including anterior, posterior, or combined approaches have been applied during the past decades. However, there is no consensus regarding the most suitable approach for thoracolumbar burst fractures with neurologic deficit [5, 6].

Decompression, stabilization, deformity correction, function preservation, and risk of complications are all important factors needed to be considered before surgery. Complete direct spinal decompression and anterior reconstruction can be achieved by anterior approaches. However, this approach is surgically more challenging and associated with significant complications including pneumothorax, aortic injury, disruption of the lumbar plexus, retrograde ejaculation, and abdominal or diaphragmatic hernia [7]. Contrastly, posterior reduction and stabilization through pedicle screw–rod instrumentation has been widely used nowadays [6, 8]. The three-column fixation characteristics of pedicle screw enables favorable stiffness and deformity correction. Meanwhile, the indirect decompression through ligaments stretch reduction, laminectomy, or partial removal of the pedicles during posterior surgery can offer comparable neurologic outcome [9, 10]. Nevertheless, it is doubtful sometimes whether an efficient canal decompression has been achieved by these indirect means during posterior surgeries, especially when the canal encroachment is caused by repulsed bone fragments from injured vertebral bodies [11] and the posterior longitudinal ligament is likely to be injured, or the intra-canal fracture fragments are located in apterium of the posterior longitudinal ligament [12]. Comprehensive meta-analysis shows an inferior canal remodeling in the indirect decompression group than the direct ones [10] and those unresolved bone fragments might cause delayed neural damage or neurologic deterioration [11, 13]. Moreover, traditional open posterior approach requires massive paraspinal muscles stripping that may lead to atrophy and contractile properties, associating with refractory postoperative back pain and disability [14]. With the increasing emphasis on protection of paraspinal muscles and posterior ligamentous complex (PLC), minimally invasive spine surgery has been introduced for the treatment of thoracolumbar fracture [15]. However, previous posterior minimally invasive technique such as the widely used percutaneous pedicle screw fixation and kyphoplasty/vertebroplasty augmentation is still confined to thoracolumbar fracture patients without neurological deficit owing to its limitation for canal decompression [15–17].

**Fig. 1:**
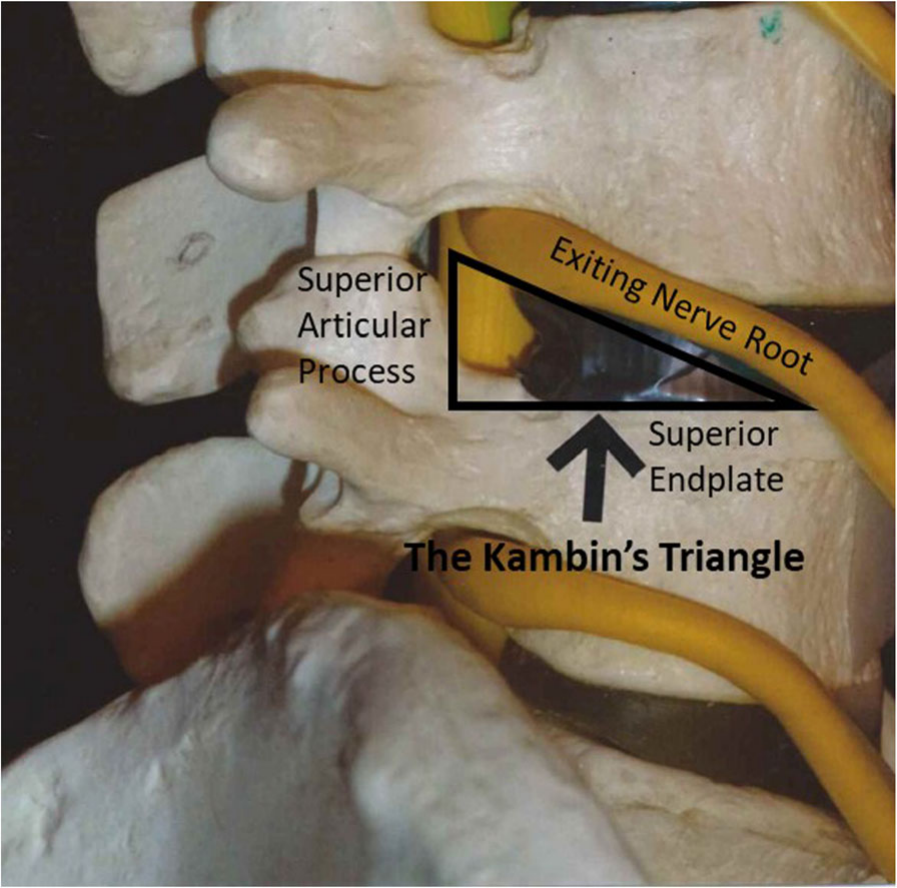
The Kambin’s Triangle, which is composed of the exiting nerve, the superior endplate of the inferior vertebra and the superior articular process, being a safe access to the disc and the spinal canal. The figure was acquired from Wikimedia Commons (http://commons.wikimedia.org/wiki/Main_Page)

As we know, the paraspinal muscle approach presented by Wiltse [18] can expose the lateral part of the facet joint and the entry point of the pedicle screw through natural intermuscular spatium without much muscle stripping. And then the Kambin’s Triangle [19] (Fig. 1), which is composed of the exiting nerve, the superior endplate of the inferior vertebra and the superior articular process, has long been used as a safe access to the disc and the spinal canal in percutaneous endoscopic lumbar discectomy (PELD) and oblique lateral lumbar interbody fusion (OLIF) surgery. As a minimally invasive way for the treatment of degenerative lumbar diseases [20], Kambin’s Triangle can provide an approach through the inferior part of intervertebral foramen directly reaching the ventral canal to achieve spinal decompression using special tools without the need for laminectomy. Based on these foundations, we investigated a new approach via the Wiltse approach reaching the Kambin’s Triangle to achieve direct decompression for the treatment of upper lumbar burst fracture with neurologic deficit. It’s technical details, clinical outcomes and complications were compared with the traditional open posterior surgery.

## Methods

### Patients

Between January 2015 and January 2016, a total of 21 consecutive patients who had traumatic upper lumbar fracture received the new approach surgery with informed consent, and they constituted the new approach group. Then we retrospectively reviewed all patients with the same problems who received the traditional open posterior surgery in our department between January 2014 and January 2015. Those who met the inclusion criteria constituted the traditional posterior surgery group. This study was approved by the Ethic Committee of the Second Military Medical University (approval number: 20140119).

The inclusion criteria were: (1) age 18–60 years; (2) admission within 7 days after injury; (3) computed tomography (CT) scans and magnetic resonance imaging (MRI) confirmed single level upper lumbar fracture (L1-L2; type A3, A4, B and C that combined with A3 or A4 according the AO classification [21]). The AO fracture classification was adopted according to Reinhold et al. [21], type C Injuries was displacement/translational injury that was characterized by displacement of cranial relative to caudal parts of the spinal column in any plane, the subtype C1 was hyperextension injury (without translation) and the subtype C2 was translation injury. We adopted the subtypes because we believe it may better reflect the severity of injury; (4) presence of neurologic deficit with canal encroachment. Patients with a history of lumbar spine surgery, severe associated injuries, old fracture, osteoporotic or pathological fracture, or other major diseases such as coagulation disorders, stroke and uremia were excluded. Patients with severe lamina fractures that have entrapped dural tissue or neural elements were also excluded.

Collected data included age, gender, injury level, AO spine classification [21], Thoracolumbar Injury Classification and Severity (TLICS) score [22]. Surgical informations including operative time, blood loss, postoperative drainage volume, postoperative hospitalization days and complications were also recorded in both groups.

For both groups, the American Spinal Injury Association (ASIA) impairment scale was used to evaluate patients’ neurologic status preoperatively, postoperatively and at 12 months follow-up. Visual Analog Score (VAS) of back pain was examined at the same times.

Radiographic parameters including the degree of canal encroachment (measured by CT), sagittal kyphosis angle and percentage of anterior height of the fractured vertebra (measured by plain radiographs) were assessed following Liao’s method [8] preoperatively, postoperatively and at 12 months follow-up. In the traditional posterior surgery group, the lamina and spinous process were removed after surgery, then the longest sagittal diameter adopted the preoperative data when measured the degree of canal encroachment postoperatively and at follow-up.

The outcome evaluation were performed by 2 independent observers who were blinded to the grouping situation. All discrepancies regarding to the TLICS, AO and ASIA classification were settled by a third observer (a senior spine surgeon).

### Surgical technique

The procedures were performed under the neurophysiological monitoring in the prone position with general anesthesia.

#### The new approach group

**Fig. 2:**
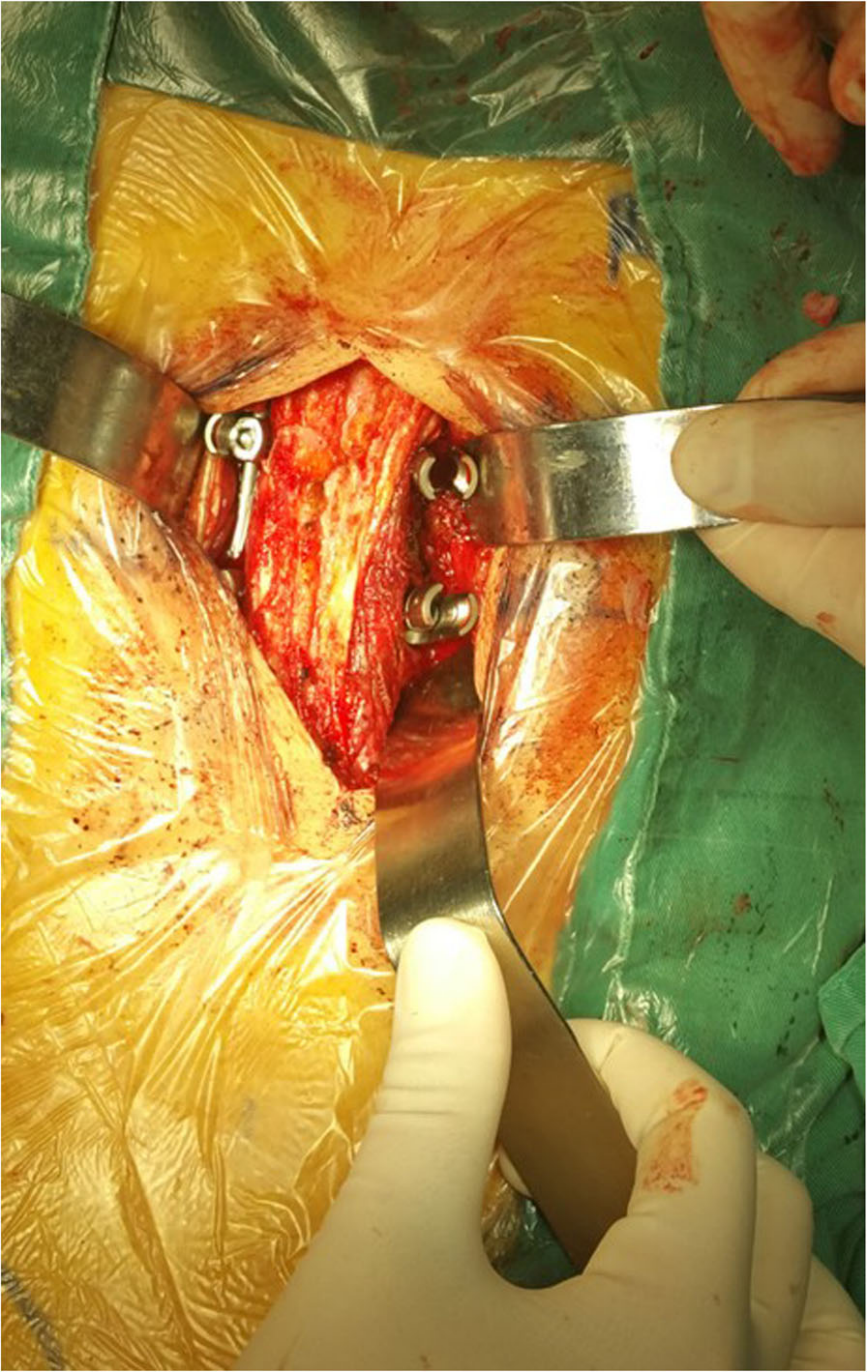
The Wiltse approach

C-arm fluoroscopy was used to locate the fractured vertebra and its adjacent vertebra above and below. A posterior midline incision was made, the integrity of supraspinous ligament and interspinous ligament were explored after the deep fascia opened. Then the intermuscular spatium between the medial multifidus and lateral longissimus muscles was bluntly separated via the Wiltse approach [18], exposing bilateral articular processes and transverse processes of the fractured vertebra, as well as its adjacent vertebras (Fig. 2). The entry point to the pedicle was identified by the intersection between the lateral border of the superior articular processes and the bisecting line of the transverse process. Pedicle screws were then inserted into the adjacent vertebra above and below the fracture. A shorter pedicle screw (30-35 mm) was inserted into bilateral or unilateral pedicles of the fractured vertebra depending on the integrity of the pedicles. The rod was prebended at a slightly lordotic angle according to the position of the fractured vertebra and connected to the screws bilaterally. Posterior distraction using the spreader forceps was performed to correct the kyphotic deformity, restore the anterior body height, and create tension of the posterior longitudinal ligament.

**Fig. 3:**
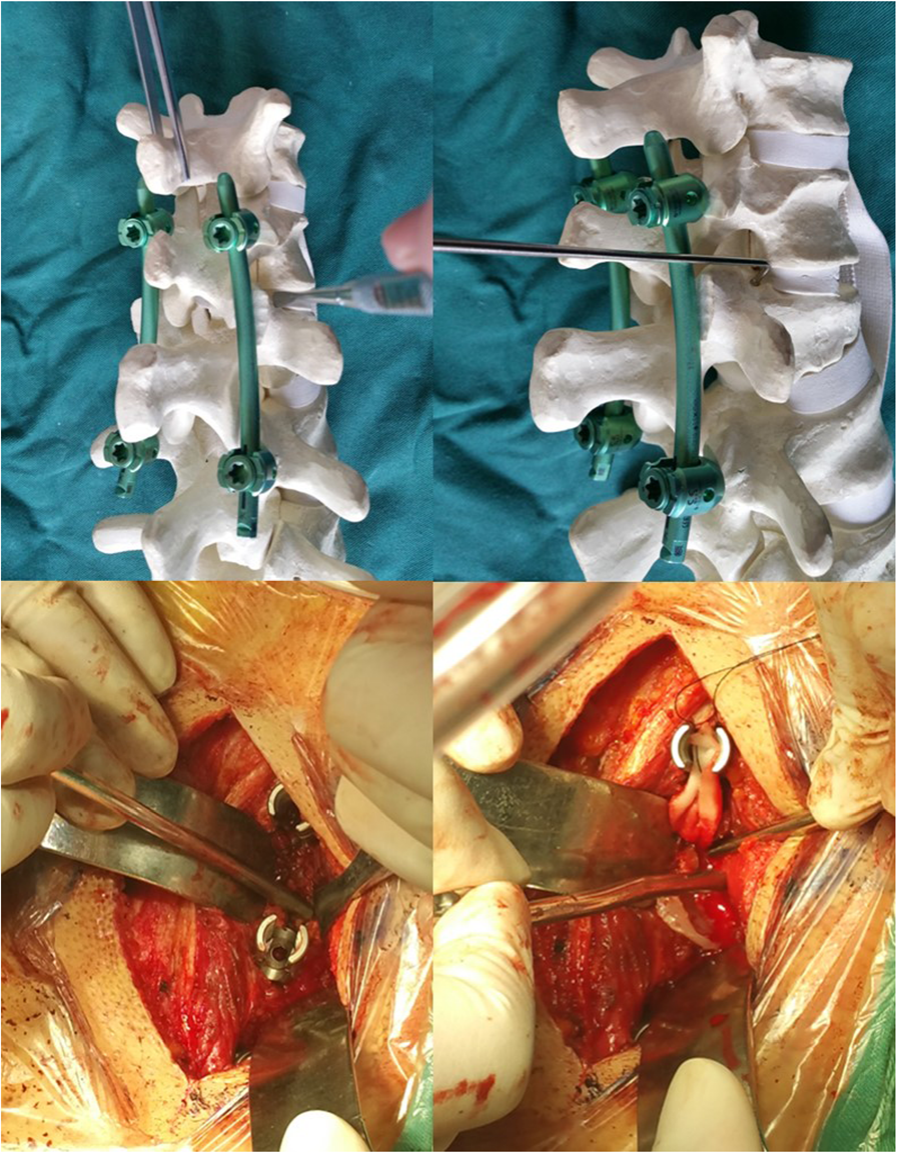
Using dura probe and stripper to explore along the lateral articular process going into the lateral wall of the canal, then reaching the front of the canal

**Fig. 4:**
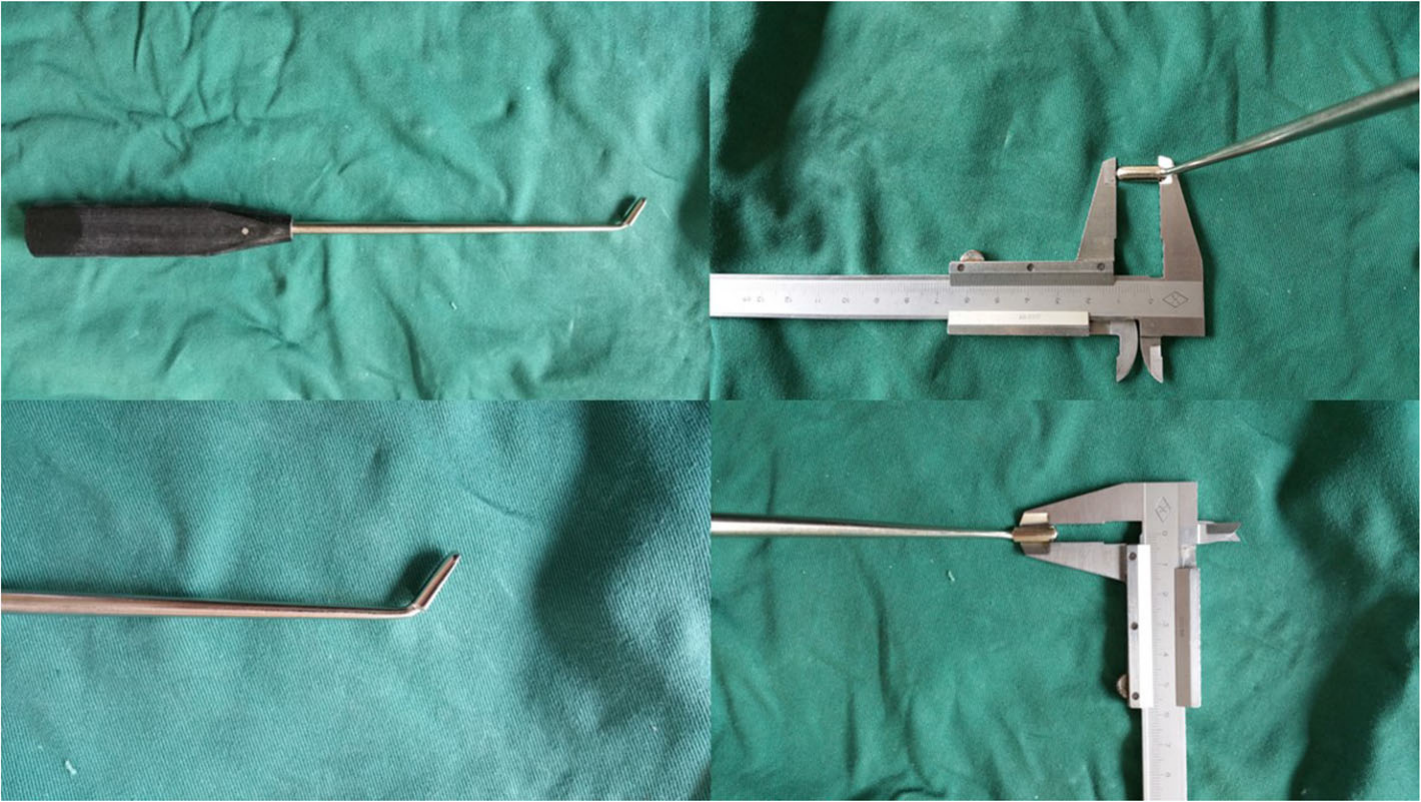
The “L” shape tamp, the angled tip is 12 mm long and 3.5 mm wide

**Fig. 5:**
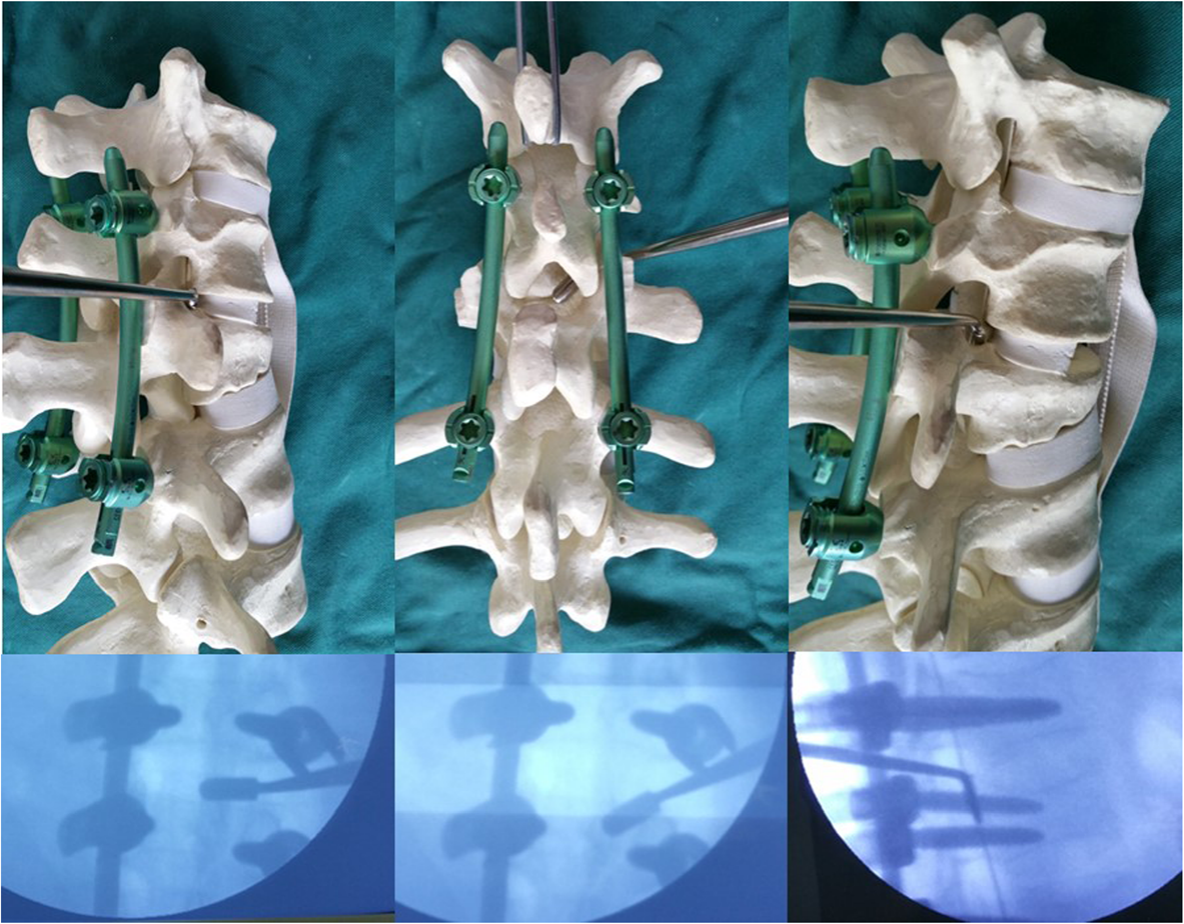
The model shows how to reduce the intra-canal fracture fragments using the “L” shape tamp and confirmed by fluoroscopy

According to preoperative symptoms, neurological signs and the position of intra-canal fracture fragments, direct spinal canal decompression through the Kambin’s Triangle was performed in the severe side at first. After temporarily remove the connecting rod, a thin stripper was used to strip the lateral superior articular process of the fractured vertebra down towards the root of transverse process, then to the front of superior articular process carefully. The soft tissue was separated to the outside and protected with brain cotton sheet. Dura probe and stripper were used to explore along the lateral articular process going into the lateral wall of the canal, then reaching the front of the canal (Fig. 3). After exploring along the pedicle and posterior wall of the fractured vertebra to confirm the position and extent of the intra-canal fracture fragments, the possible adhesion between the fracture fragments and the ventral dura was carefully separated Then an “L” shaped tamp (Fig. 4) with appropriate length and angle was inserted into the canal along the exploratory route. After slightly adjusted the “L” shaped tamp to allow the angled tip move onto the intra-canal fracture fragments and below the ventral dura, we put pressure gradually and repeatedly to reduce the intra-canal fracture fragments and facilitate complete reduction (Fig. 5). The lateral view of the fluoroscopy was used to confirm if adequate reduction of the fracture was achieved (Fig. 5). Then the connecting rod was fixed again. Same procedure was repeated on the other side if necessary. In some cases, the anterior part of the superior articular process and the superior part of the vertebral pedicle could be partly removed to modify the intervertebral foramen in order to facilitate the decompression and reduce the stretching of nerve root. No laminectomy or bone graft fusions was performed. The supraspinous ligament or interspinous ligament was repaired by suture when ligament injury existed.

**Fig. 6:**
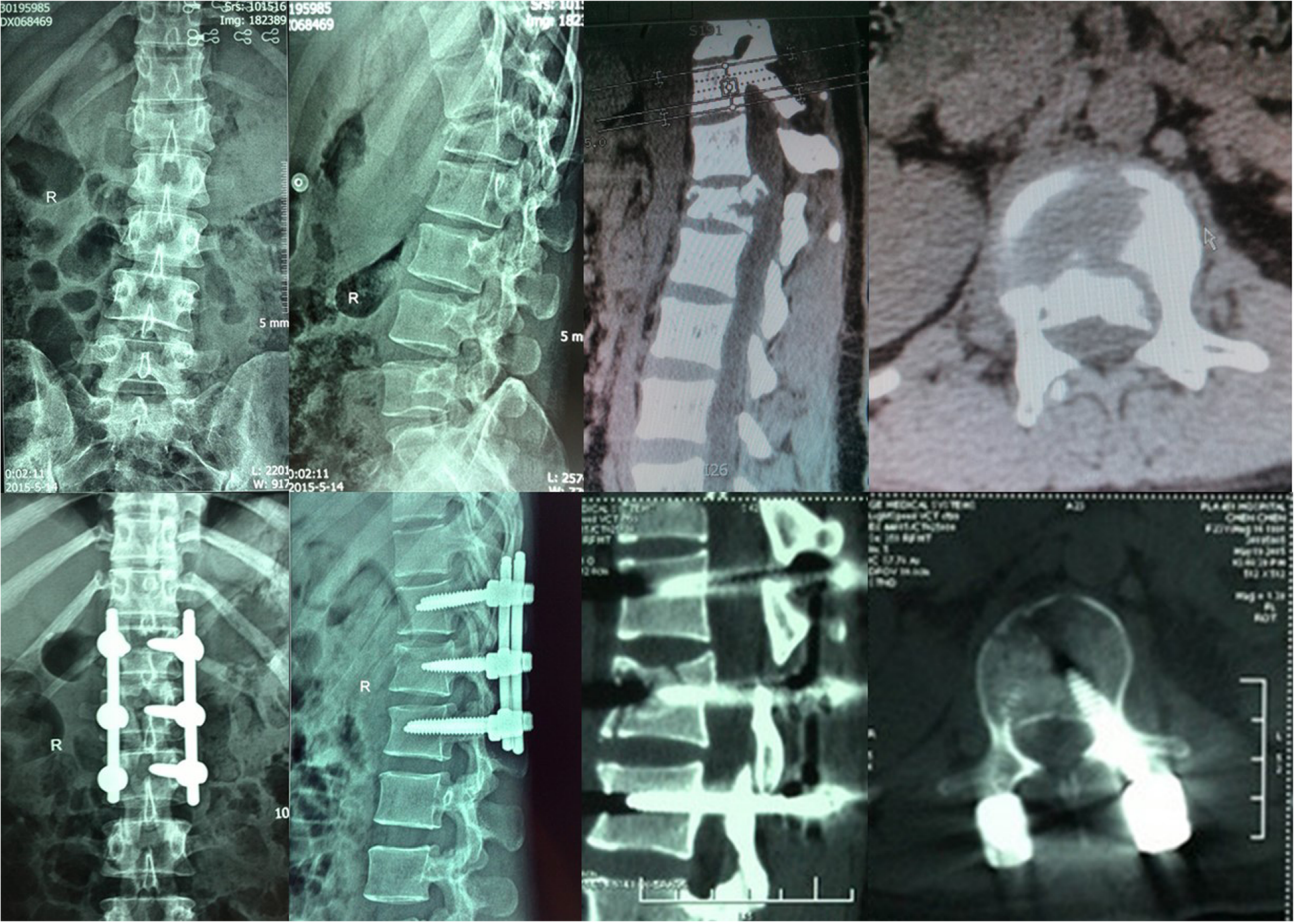
A 23 years old female with L1 burst fracture treated by the present new approach surgery. Preoperative and postoperative X-ray and CT are shown. The canal encroachment were efficiently relieved, as well as the sagittal kyphosis angle and percentage of anterior height of the fractured vertebra were all significantly improved postoperatively

#### The traditional posterior surgery group

The traditional posterior surgery was the posterior midline approach and was performed as Kong et al. [11] described.

#### Postoperative management

The postoperative management was uneventful. Patients were encouraged to ambulate with a hard brace in 5 days postoperatively and the hard brace was kept for 3 months. All the included patients were followed for at least 12 months. Instrumentation would be taken out approximately 12 months after surgery in both groups.

### Statistical analysis

Statistical analysis was performed with SPSS 21.0 (SPSS, Chicago, Illinois, USA). Continuous variables were compared by using a t-test. Categorical variables were compared by using χ2 test or Fisher’s exact test. We assigned one point for each grade recovery to quantify the neurologic recovery index for the two groups. The significance level was set at 0.05.

## Results

In total, 21 patients were included in the new approach group and 30 patients received the traditional open posterior surgery. All patients were operated successfully and no major complications such as deep wound infections, neurologic deterioration, hardware failure or pseudarthrosis were reported. One patient in the traditional posterior surgery group lost the follow-up. No statistical significant difference was detected concerning the demographic data between these two groups (*P* > 0.05) (Table 1). However, patients in the new approach group had fewer operation time (128.3 ± 25.1 vs 151 ± 32.2 min, *P* = 0.01), less blood loss (243.8 ± 135.5 vs 437.8 ± 224.9 ml, *P* = 0.001) and drainage volume (70.7 ± 57.2 vs 271.7 ± 95.5 ml, *P* < 0.001), as well as shorter hospitalization stay than the traditional posterior surgery group (6.6 ± 1.8 vs 8.5 ± 2.4 d, *P* = 0.004) (Table 2).

**Table 1:** Demographic Data

	New approach group	Traditional posterior surgery group	P
Cases	21	29	
Age(y)	37.7 ± 10.9	39.5 ± 9.7	0.54
Male	16	20	0.574
Injured Level			0.917
L1	12	17	
L2	9	12	
AO fracture classification			0.974
A3	5	6	
A4	8	9	
B2	3	6	
C1	3	5	
C2	2	3	
TLICS score	6.5 ± 1.9	6.8 ± 2	0.542

TLICS, Thoracolumbar Injury Classification and Severity

The AO fracture classification was adopted according to Reinhold et al. [21]

**Table 2:** Perioperative Data

	New approach group(*n* = 21)	Traditional posterior surgery group(*n* = 29)	P
Operation time(min)	128.3 ± 25.1	151 ± 32.2	0.01
Blood loss(ml)	243.8 ± 135.5	437.8 ± 224.9	0.001
Drainage volume(ml)	70.7 ± 57.2	271.7 ± 95.5	<0.001
Hospitalization stay(d)	6.6 ± 1.8	8.5 ± 2.4	0.004

All the radiographic parameters had evident improvement postoperatively and at 12 months follow-up in both groups (*P* < 0.05) (Table 3). Though the preoperative canal encroachment, kyphosis angle and anterior height were not significantly different in the two groups (*P* > 0.05), the canal encroachment were better relieved in the new approach group (4 ± 3.8 vs 9.1 ± 6, P = 0.001) (Table 3).

**Table 3:** Radiographic Parameters

	New approach group(n = 21)	Traditional posterior surgery group(n = 29)	P
Canal encroachment(%)			
Preoperation	45.6 ± 17.7	47.5 ± 19.8	0.729
Postoperation	6.1 ± 5.4	13.1 ± 7.5	0.001
Last follow-up	4 ± 3.8	9.1 ± 6	0.001
Kyphosis angle(°)			
Preoperation	21.1 ± 3.1	22.3 ± 3.3	0.2
Postoperation	6.1 ± 2.2	5.1 ± 1.2	0.072
Last follow-up	9.3 ± 2.6	10.3 ± 2.4	0.171
Anterior height(%)			
Preoperation	53.2 ± 11.8	51.5 ± 12.1	0.633
Postoperation	90.1 ± 7.9	88.4 ± 9.2	0.51
Last follow-up	88.2 ± 8.4	86.3 ± 10.1	0.487

As to the pain relief and neurologic recovery (Table 4), both groups had significant improvement in VAS and ASIA impairment scale postoperatively and at 12 months follow-up (*P* < 0.05). The new approach group achieved better outcome in VAS postoperatively and at 12 months follow-up than the traditional posterior surgery group (2.6 ± 0.7 vs 3.5 ± 0.9, *P* < 0.001 and 1.4 ± 0.9 vs 2.4 ± 0.8, P < 0.001). However, no significant difference was found in neurologic recovery between the two groups at 12 months follow-up (*P* > 0.05). According to the ASIA grade of neurologic recovery, 15 patients achieved one grade recovery, 2 had two grade recovery, and 4 remained the same in the new approach group; while 21 patients achieved one grade recovery, 2 gained two grade recovery, and 6 remained the same in the traditional posterior surgery group. There was no significant difference in neurologic recovery index between the two groups (0.90 ± 0.53 vs 0.86 ± 0.51, *P* = 0.778), indicating that the neurologic recovery in the new approach group is similar to the traditional posterior surgery group.

**Table 4:** American Spinal Injury Association Impairment Scale and Visual Analog Score

	New approach group(*n* = 21)	Traditional posterior surgery group(*n* = 29)	P
VAS			
Preoperation	7.9 ± 0.8	7.7 ± 1	0.45
Postoperation	2.6 ± 0.7	3.5 ± 0.9	<0.001
Last follow-up	1.4 ± 0.9	2.4 ± 0.8	<0.001
ASIA			
Preoperation			0.706
A	0	2	
B	3	6	
C	7	8	
D	11	13	
Last follow-up			0.963
A	0	1	
B	1	2	
C	3	6	
D	6	7	
E	11	13	
Recovery index	0.90 ± 0.53	0.86 ± 0.51	0.778

ASIA, American Spinal Injury Association Impairment Scale

VAS, Visual Analog Score

## Discussion

Although thoracolumbar burst fractures are common, the optimal therapeutic option still remains challenging [5, 6]. For neurologically intact patients without spinal instability, nonoperative treatment including short-term bed rest, brace or orthosis could achieve favorable results [23]. The TLICS score proposed by Vacarro et al. has been widely used as a treatment algorithm for clinical decision making in thoracolumbar fractures [22]. This novel classification considers 3 primary parameters including the fracture morphology, the neurologic status and the integrity status of posterior ligamentous complex (PLC) with a total of 10 points to determine stability and treatment option. According to this classification, nonoperative treatment is recommended for patients with a score less than 4. However, a quarter of initially conservatively treated patients might turn to surgery due to disabling pain [24]. Moreover, the posttraumatic kyphotic deformity and back pain might progress after long term follow-up in nonoperatively treated patients despite adequate bracing [6]. For thoracolumbar burst fractures with neurologic deficit, the TLICS score reached minimally 4 points and operative treatment is recommended. More recently, by combining the key benefits of TLICS and AO-Magerl classification, the AOSpine Classification Group proposed the AOSpine Thoracolumbar Spine Injury Classification System that simultaneously consider the morphological description of spinal column injuries, all major modes of failure and clinical features such as neurological status and treatment modifiers [21]. And it may serve as a more valuable tools for communication, patient care, and research purposes in the future. In order to stabilize the spine, recover sagittal balance, decompress neural elements and obtain early patient mobilization, various surgical procedures including anterior, posterior, or combined approaches have been applied in the treatment of thoracolumbar burst fractures with neurologic deficit. However, there is still no consensus regarding the most suitable approach currently [5, 6].

Anterior approaches can achieve complete removal of the retropulsed bone and soft tissue fragments from the spinal canal under direct visualization without manipulation of the dural tube, as well as anterior reconstruction using plate or rod with bone graft [25]. Theoretically, the anterior approach offers a satisfactory canal decompression and a better chance of neurologic improvement compared to other procedures [25]. However, this approach is surgically challenging and more likely to have complications due to the adjacent chest and abdominal organs as well as major blood vessels [7]. For example, Lin et al. [26] reported that patients in the anterior approach group had 5 times more complications but similar neurologic improvement comparing to the posterior approach group.

On the other hand, posterior pedicle screw–rod instrumentation could provide favorable stiffness and deformity correction due to its three-column fixation characteristics [9]. Posterior approach with pedicle screw–rod instrumentation has been widely used for most thoracolumbar fractures nowadays [6, 8] since it can achieve favorable outcomes in terms of spine stabilization, kyphosis correction, postoperative neurologic improvement [9, 10, 26]. However, traditional open posterior operation requires massive paraspinal muscles stripping to expose the spinous process, lamina and facet, followed by short-segment (1 level above and below the injured level) or long-segment (2 levels above and below the injured level) internal fixation. The laminectomy is also performed at the injured level for canal decompression in patients with neurologic deficit. In this approach, massive paraspinal muscles stripping would cause ischemia, necrosis and denervation of the paraspinal muscle, resulting in atrophy and contractile properties loss of paraspinal muscles postoperatively. Denervation and dysfunction of the paraspinal muscles, as well as destruction of the posterior column stability, are believed to be associated with refractory postoperative back pain and disability [14]. Recently, Li et al. [27] measured the cross-sectional area of the paraspinal muscle using MRI to compare the paraspinal muscle between the minimally invasive transforaminal lumbar interbody fusion (miTLIF) group and the traditional open TLIF group after the treatment of 1-segment lumbar disease. After 48 months follow up, patients in the traditional open TLIF group had significantly smaller cross-sectional area of the paraspinal muscle, complicated with worse back pain VAS scores and ODI scores, indicating the advantages of miTLIF in preventing paraspinal muscle atrophy, reducing postoperative back pain and improving postoperative life quality [27]. Another disadvantage of the traditional posterior approach is that the canal decompression might be limited, especially when the canal encroachment is caused by repulsed bone fragments from injured vertebral bodies [11] and the posterior longitudinal ligament is likely to be injured, or the intra-canal fracture fragments are located in apterium of the posterior longitudinal ligament [12]. Although some authors have reported no significant association between the extent of canal encroachment and neurological function [28, 29], a complete canal decompression theoretically offers a better chance for neurologic improvement and a more complete canal decompression with low risk of complications is worth trying for patients with neurologic deficit.

In this study, we presented a new approach via the Wiltse approach and the Kambin’s Triangle to achieve rigid fixation and direct decompression for the treatment of upper lumbar fracture with neurologic deficit. Compared to the traditional open posterior approach, this new approach has the following advantages: (1) The Wiltse approach enables pedicle screw implantation through natural intermuscular spatium without much muscle stripping and injury, thus preventing paraspinal muscle atrophy and reducing postoperative back pain. This was confirmed in our study that the pain level in the new approach group was significantly lower postoperatively and at 12 months follow-up. (2) The new approach can reduce intraoperative blood loss because major blood vessels are avoided through the natural intermuscular spatium. (3) The new approach is able to reach the ventral canal directly via the Kambin’s Triangle without the need for laminectomy, and the “L” shaped decompressor is applied to put pressure directly onto the intra-canal fracture fragments for reduction. Thus, the decompression becomes more efficient and complete without implicating the dura and neural elements. The average operation time was over 20 min shorter in the new approach surgery due to the avoidance of laminectomy. CT showed that canal encroachment was better relieved postoperatively and canal remodeling was more satisfied achieved at 12 months follow-up in the new approach group. Although neurologic recovery in the new approach group is similar to traditional posterior surgery group, the prior approach offers a more complete canal decompression and a theoretically better chance for neurologic improvement with shorter operation time and less blood loss. Moreover, if the intra-canal fracture fragments can’t be reduced by the decompressor, operators would get a feedback and laminectomy can therefore be performed as a remedy for decompression. The Kambin’s Triangle is a safe access to the disc and the spinal canal [19] and it has long been used in PELD and OLIF surgery as a minimally invasive technique for the treatment of degenerative lumbar diseases [20]. In addition, partly removal of the anterior part of the superior articular process would not increase the risk of segmental instability [30], and can be performed in some cases to reduce nerve root stretching and facilitate decompression. (4) The present new approach can keep the integrity of posterior ligamentous complex (PLC) since it cause no further injury to the PLC, and the supraspinous ligament or interspinous ligament could be repaired by suture when ligament injury existed [31].

For severe unstable thoracolumbar burst fractures, posterior short-segment fixation alone without anterior support might result in implant failures. To determine certain fractures that would require supplemental anterior reconstruction, McCormack et al. [32] proposed the load sharing classification (LSC) determined by three components including comminution of the fractured body, apposition of fragments and kyphosis correction with a total of 9 points. According to McCormack et al., anterior reconstruction was necessary for patients with an LSC ≥ 7 because posterior short-segment fixation alone would lead to implant failures. Nevertheless, several techniques have been proposed to avoid complicated anterior surgeries and prevent implant failures in posterior surgeries for severe unstable thoracolumbar burst fractures (LSC ≥ 7) [33]. The application of intermediate screws at the level of the fracture could increase the stiffness of the posterior short-segment construct and protect the anterior column during loading [34], avoiding the need for anterior reconstruction in the treatment of severe unstable thoracolumbar burst fractures (LSC ≥ 7) [33, 35]. Therefore, in the present new approach surgery, we preferred to insert bilateral or unilateral intermediate screws into the fractured vertebra depending on the integrity of the pedicles in order to increase fixation strength and facilitate reduction. A shorter intermediate screw (30-35 mm) was competent because pedicle contributes approximately 80% of the stiffness and 60% of the pullout strength at the screw-bone interface [33]. Also, the Wiltse approach could provide broad operative field that was easy for implantation of pedicle screws and the prebent connecting rods. Our results showed a good reduction and kyphosis correction without implant failures in all the included patients through the posterior short-segment fixation with intermediate screws.

Limitations for current new approach should be noticed as well. Firstly, the present new approach is limited to only a portion of upper lumbar fractures. Thoracic spine fractures or patients with severe lamina fractures that have entrapped dural tissue or neural elements are not applicable. Secondly, its application relies on the special “L” shape tamps with various lengths and angles to adapt to different conditions according to each case. Thirdly, complications such as epidural veins bleeding and cerebrospinal fluid leakage during canal decompression are difficult to control in the present new approach, and open surgical technique may be needed in these conditions. Fourthly, as an innovative surgery, it has a learning curve and the beginners should be trained for a period of time, starting from some simple cases, such as burst fracture without large spinal canal compression. Lastly, more cases with longer follow-up are needed because current sample size and follow-up time are not adequate enough to draw a valid conclusion (Fig. 6).

## Conclusions

The present new approach was successfully applied in the treatment of upper lumbar fracture with neurologic deficit. Though with some limitations, it was superior in reducing the iatrogenic trauma while achieving similar or even better clinical and radiological outcomes compared to the traditional posterior surgery.

## Data Availability

The datasets used or analysed during the current study are available from the corresponding author on reasonable request.

## Abbreviations

ASIA: American spinal injury association
CT: Computed tomography
LSC: Load sharing classification
MRI: Magnetic resonance imaging
OLIF: Oblique lateral lumbar interbody fusion
PELD: Percutaneous endoscopic lumbar discectomy
PLC: Posterior ligamentous complex
TLICS: Thoracolumbar injury classification and severity
VAS: Visual analog score
